# Renal vein thrombosis in the post-partum period: a case report

**DOI:** 10.1186/1752-1947-8-462

**Published:** 2014-12-24

**Authors:** Abdelilah Mouhsine, Ahmed Belkouch, El Mehdi Atmane, Redouane Rokhssi, Youssef Berrada, Lahcen Belyamani, Mbarek Mahfoudi, Abdelghani El Fikri

**Affiliations:** 32 Department of Radiology, Avicenna Military Hospital, Avenue Al Mouqaouama, Marrakech, 40000 Morocco; 33 Emergency Department, Mohamed V Military Hospital of Instruction, Hay ryad avenue des Far, Rabat, 10000 Morocco

**Keywords:** Flank pain, Hematuria, Hypercoagulability, Post-partum, Venous renal infarction

## Abstract

**Introduction:**

Physiological hypercoagulability is a known condition in pregnancy designed to limit the risk of bleeding; it may exceptionally be complicated by thrombosis of the renal vein. To the best of our knowledge, this is the third case of renal venous infarction reported in the literature.

**Case presentation:**

We report the case of a 43-year-old Caucasian woman, a mother of three sons who presented with left flank pain and hematuria. The clinical investigations did not find any other cause for her thrombophilia.

**Conclusions:**

Clinical onset is not specific, so it is important to evoke the diagnosis in the context of pregnancy; computed tomography angiography is the investigation of choice to set the diagnosis. It is important to know that anticoagulation therapy must be initiated as soon as possible.

## Introduction

Renal vein thrombosis is relatively rare; a pregnant woman has a relative risk of vein thrombosis higher than non-pregnant women. This condition leads exceptionally to renal vein infarction [1]. The clinical presentation is non-specific, it can be insidious or severe, and computed tomography (CT) angiography is the cornerstone to diagnosis.

## Case presentation

A 43-year-old Caucasian woman, a mother of three sons, at 30 days post-partum after a full-term pregnancy without any complications and concluded by a vaginal delivery, suffering from diabetes on oral antidiabetic medication, presented to the emergency department with left flank pain with hematuria of one week’s duration. A clinical examination revealed an alert patient, febrile at 38°C, with blood pressure of 158 over 67mmHg, heart rate of 90 beats per minute, respiratory rate of 20 breaths per minute, suffering from pain in the left flank (visual analog score (VAS) = 5), this pain was exacerbated by palpation, which found lumbar contact. Diagnostic studies in the emergency room revealed a urine analysis with 104 white blood cells (WBCs), gross hematuria, and a C-reactive protein level of 88. Her blood cell count revealed anemia at 9g of hemoglobin; blood electrolytes and renal function with serum creatinine levels were normal.

**Figure 1:**
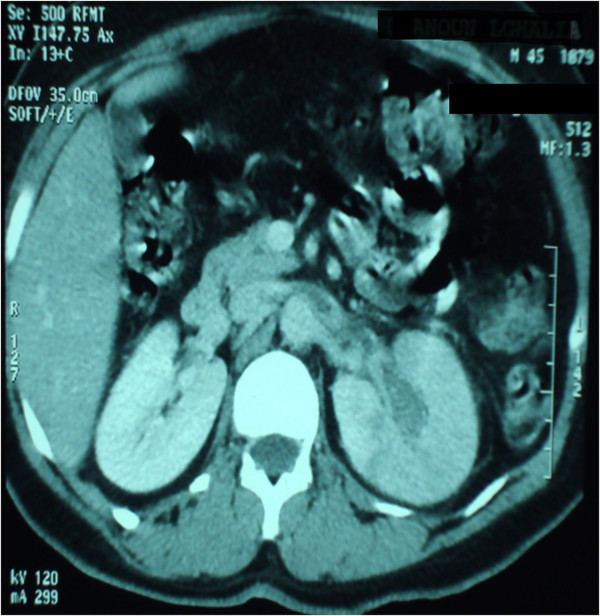
**Computed tomography axial section after injection of contrast medium in parenchymal time showing a large thrombus in the left renal vein extending from the segmental renal veins to the inferior vena cava, complicated by cortical infarction.**

**Figure 2:**
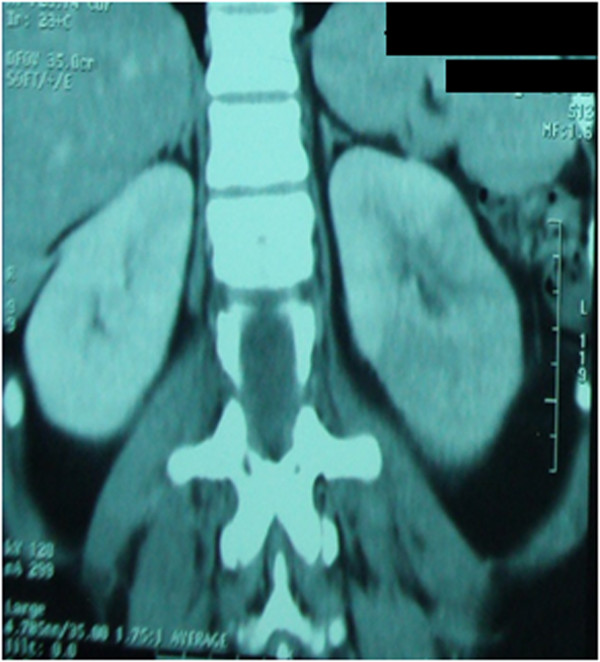
**Computed tomography scan after injection of contrast medium in parenchymal window showing infarction of the lower pole of the left kidney.**

An abdominal ultrasound scan showed discrete pyelocalyceal cavities dilation with a slightly enlarged left kidney and without visible urinary obstruction. A CT urography and angiography scan was performed and showed an ectatic left renal vein, seat of a large hypodense thrombus extending from the segmental renal veins to the inferior vena cava, with a large heterogeneous and hypodense region located in the medium and lower poles of the left kidney without urinary tract dilatation or tumoral lesions; this aspect concluded in a perfusion abnormality (renal venous infarction) (Figures 1 and 2).

Anticoagulation heparin therapy was started and our patient was admitted to a medical center for further investigations. Her proteinuria and blood cultures did not show any abnormalities, and her serum albumin levels were normal. The thrombophilia assessment, with protein C or S deficiency, mutation of coagulation factors II and V, lupus and antiphospholipid antibody syndrome (APS) tests, were negative. The outcome was favorable with disappearance of pain and, after five days of hospitalization, our patient was put on oral anticoagulant therapy (acenocoumarol 4mg/day) and discharged.

## Discussion

The constitution of venous thrombosis according to Virchow requires the combination of three elements: hypercoagulability, slowing down of the blood flow (stasis) and endothelial lesion. However, a single factor may be sufficient for the occurrence of this abnormality [2].

Physiological hypercoagulability is a known condition in pregnancy and the post-partum period aimed to limit the risk of bleeding at the time of delivery [3, 4]. Hypercoagulability induced by hormones and mechanical compression by the gravid uterus are the two mechanisms evoked to explain this physiological condition. Normal pregnancy is also associated with increased levels of coagulation factors, VII, VIII, X, Von Willebrand factor, and high concentrations of fibrinogen. In addition, we note low levels of anticoagulant proteins: protein S association with the complement C4b is higher than for nonpregnant women, also antithrombin III is partially evacuated by the urine [5, 6].

A pregnant woman has a relative risk of thrombosis five to six times higher than nonpregnant women of the same age [7]. However, the absolute risk of venous thromboembolism (VTE) remains low. McColl *et al.* [8], in a retrospective series including 72,201 births in Glasgow from 1985 to 1996, reported an incidence of VTE, proven on objective tests of 0.71 per thousand for the deep vein thrombosis (DVT) and 0.15 per thousand for pulmonary embolism.

Symptoms of renal vein thrombosis can occur insidiously with nonspecific signs such as a single lower limb edema. The onset can also be acute with associating flank pain, gross hematuria, anemia, fever and proteinuria; this clinical picture can rapidly progress to kidney failure if thrombosis is bilateral or if it occurs in a single kidney [9]. This vein thrombosis may occur in a context of infection especially post-abortion. In this situation, a search for an infectious thrombophlebitis is required. The search for thrombophilia, particularly under APS, should be active.

The paucity of clinical signs and the absence of laboratory tests for confirmation make radiology an essential step in the diagnosis. In the acute phase, an ultrasound scan will show hyperechogenic and enlarged kidneys in 90% of cases [10]. The color Doppler ultrasound is not very effective for detecting the segmental thrombosis, but is commonly used to detect fresh thrombosis in patients undergoing renal transplantation [11]. The CT angiography, with a sensitivity and specificity approaching 100% [12], remains the investigation of choice by directly visualizing the thrombus or by the detection of indirect signs, such as increased renal size, dilated renal vein, delay, reduction or absence of opacification of the collecting system, a persistent nephrogram attributable to poor venous drainage; delayed corticomedullary differentiation, and thickening of the renal fascia [12].

Magnetic resonance imaging (MRI) is a good alternative to CT angiography, especially to avoid the use of nephrotoxic contrast material. However, its sensitivity and specificity are lower than CT angiography [13].

Anticoagulant heparin therapy should be initiated early to prevent the spread of the thrombus. They should be relayed with oral anticoagulation after three to 10 days and must be taken long term. The objective is to have the international normalized ratio (INR) in the range between two and three.

For our patient, the etiological research did not reveal other causes of thrombophilia and in the absence of an infectious or tumor etiology, anticoagulation therapy alone has been initiated and decided to be maintained for one year, and the outcome has been favorable to date. To the best of our knowledge, this is the third case of spontaneous post-partum renal venous infarction after the one described by Mansi [1].

## Conclusions

Physiological hypercoagulability is a known condition in pregnancy, and may very rarely be complicated by thrombosis of the renal vein. Due to the non-specific clinical picture, the practitioner must be aware of such a diagnosis and should consider it in pregnant and post-partum women. Cross-sectional imaging, particularly a CT scan with intravenous contrast, is the gold standard for diagnosis, as it provides a comprehensive picture and is useful for the posttherapeutic assessment.

## Consent

Written informed consent was obtained from the patient for publication of this case report and any accompanying images. A copy of the written consent is available for review by the Editor-in-Chief of this journal.
